# Recombinant canstatin inhibits VEGF‐A‐induced lymphangiogenesis and metastasis in an oral squamous cell carcinoma SCC‐VII animal model

**DOI:** 10.1002/cam4.866

**Published:** 2016-09-20

**Authors:** Jeon Hwang‐Bo, Jong‐Hwa Park, Mun Gyeong Bae, In Sik Chung

**Affiliations:** ^1^ Department of Genetic Engineering and Graduate School of BiotechnologyKyung Hee UniversityYongin17104Korea

**Keywords:** Canstatin, lymphangiogenesis, oral squamous cell carcinoma, SCC‐VII, VEGF‐A

## Abstract

We describe the inhibitory effects of recombinant canstatin on tumor growth and lymphangiogenesis induced by an oral squamous cell carcinoma (SCC) using an orthotropic oral SCC animal model. Recombinant canstatin treatment decreased final tumor volumes and weights, as well as densities of blood and lymphatic vessels. Lung metastasis of oral SCC was significantly reduced in recombinant canstatin‐treated animals. Recombinant canstatin reduced vascular endothelial growth factor (VEGF)‐A expression in SCC‐VII cells treated with the hypoxia mimetic agent, CoCl_2_. VEGF‐A induced in vivo lymphatic vessel formation in a Matrigel plug, but this was remarkably reduced in a recombinant canstatin‐treated Matrigel. Recombinant canstatin suppressed the expression of vascular endothelial growth factor receptors (VEGFR)‐1 and ‐2 stimulated by VEGF‐A. Based on immunohistochemical analysis, recombinant canstatin significantly reduced the expression of VEGF‐A, VEGFR‐1, and ‐2 in SCC‐VII‐induced tumors. Recombinant canstatin did not affect the expression of VEGF‐C or VEGFR‐3. In addition, recombinant canstatin suppressed the VEGF‐A‐induced phosphorylation of VEGFR‐1 and ‐2. Our results indicate that recombinant canstatin exhibits antitumoral and antilymphangiogenic activities against oral SCC cells. Antilymphangiogenic signaling by recombinant canstatin is probably mediated by the suppression of the integrin *α*v*β*3/VEGFR‐1 and/or ‐2 signaling induced by VEGF‐A. Our results also suggest that recombinant canstatin has a high potential to inhibit oral SCC‐induced tumors and lymphatic metastasis.

## Introduction

Head and neck cancers are comprised of a variety of malignant tumor types that occur in the head and neck region. Squamous cell carcinoma (SCC), also known as squamous cell cancer, is the most common type of malignant tumor of the head and neck region and represents more than 90% of all head and neck cancers. Head and neck squamous cell carcinoma (HNSCC) ranks sixth as the most common cancer worldwide. HNSCC treatment has changed over the last 20 years with the introduction of new approaches, including combined modality therapy and improvements in surgery and radiotherapeutic procedures 1. However, HNSCC patient survival rate has not improved significantly due to continued high local and distant failure rates and occurrence of second primary tumors. Therefore, demand for new HNSCC treatment methods remains unabated.

Lymphangiogenesis, which is the process of new lymphatic vessel development from existing lymphatic vessels, plays an important role in many physiological and pathological processes, such as embryonic development, organ transplantation, wound healing, regeneration of tissues and organs, and tumor metastasis 2. The spreading of lymphatic system‐mediated tumor cells to lymph nodes is a common occurrence and is an early event in diseases involving metastasis 3. The lymphatic system is of greater importance than the vascular system in metastasis of HNSCC. Therefore, inhibition of tumor lymphangiogenesis is a target for therapies intended to prevent cancer metastasis 4. Tumor‐induced lymphangiogenesis is caused by tumors expressing lymphangiogenic factors, including vascular endothelial growth factor (VEGF) family members, fibroblast growth factor (FGF), angiopoietin‐1 and ‐2, and platelet‐derived growth factors (PDGFs) 5, 6. VEGF‐C and ‐D are predominant lymphangiogenic factors that induce lymphangiogenesis through vascular endothelial growth factor receptor (VEGFR)‐3 activation. VEGFR‐3 is a receptor tyrosine kinase that is expressed on lymphatic endothelial cells (LECs) 7, 8, 9. VEGF‐A has been regarded as an angiogenic factor. However, several recent studies revealed VEGF‐A inducement of angiogenesis, lymphangiogenesis, and lymphatic metastasis 10, 11.

Canstatin is a 24‐kDa peptide derived from human basement membranes. It has been identified as a fragment originating from the globular C‐terminal noncollagenous domain (NC1) of the collagen type 4 *α*2 chain 12. Canstatin inhibits tumor growth and angiogenesis in mouse models as well as significantly inhibits tube formation and migration of endothelial cells 13. Canstatin also potently inhibits 10% fetal calf serum‐induced proliferation of endothelial cells, and acts to induce apoptosis. Additionally, it inhibits Fas‐dependent apoptosis and Akt activation in endothelial cells 14. In our previous studies, recombinant canstatin produced from stably transfected *Drosophila melanogaster* S2 cells inhibited the growth of tumors in orthotropic AT‐84 oral SCC and heterotropic CT‐26 colon carcinoma animal models 15, 16.

Herein, we investigated the suppressive effects of recombinant canstatin against lymphangiogenesis through in vitro experiments using SCC‐VII and human lymphatic microvascular endothelial cells (HLMECs). We also investigated the inhibitory effects of recombinant canstatin against tumor growth and lymph node metastasis using an orthotropic SCC‐VII oral SCC animal model. Our results showed that recombinant canstatin inhibits lymphangiogenesis and lymphatic metastasis via suppression of VEGF‐A/VEGFR‐1 and ‐2 signaling.

## Materials and Methods

### Cell lines and culture

Mouse SCC‐VII cells, obtained from Dr. Han‐Sin Chung of Samsung Medical Center in Seoul, Korea, were maintained in Roswell Park Memorial Institute‐1640 medium (Thermo Scientific HyClone, Logan, UT) supplemented with 10% (v/v) heat‐inactivated fetal bovine serum (FBS; Thermo Scientific HyClone) in a 5% CO_2_ humidified incubator at 37°C. Primary HLMECs (Lonza, Basel, Switzerland) were maintained in microvascular endothelial growth medium (EGM‐2 MV; Lonza) with 20% (v/v) human serum (Lonza) in a 5% CO_2_ humidified incubator at 37°C.

### Preparation of purified recombinant canstatin

Recombinant canstatin was expressed in *D. melanogaster* S2 cells stably transfected with a plasmid containing human canstatin cDNA using the lipofectamine method 17. Recombinant canstatin was subsequently purified to homogeneity using a simple one‐step Ni‐NTA affinity fractionation, as described previously 17.

### RT‐PCR analysis

SCC‐VII cells grown for 24 h in 100 cm^2^ culture dishes at a seeding density of 1.0 × 10^6^ cells/dish were treated with 0, 0.5, and 40 *μ*g/mL recombinant canstatin in the presence of 100 *μ*mol/L CoCl_2_ and incubated for an additional 24 h. HLMECs cultured for 24 h in 100 cm^2^ culture dishes at a seeding density 1.0 × 10^6^ cells/dish were treated with 0, 0.5, and 40 *μ*g/mL recombinant canstatin in the presence of 20 ng/mL recombinant human VEGF‐A (rhVEGF‐A; R&D Systems, Minneapolis, MN) and incubated for an additional 24 h. Subsequent to washing with PBS, Trizol reagent (Invitrogen, Carlsbad, CA) was used for the extraction of total RNA following the manufacturer‐supplied protocol. Two micrograms of total RNA were treated with amplification‐grade RNase‐free DNase I (Invitrogen) and used for cDNA synthesis using an Improm‐II^™^ Reverse Transcription System (Promega, Madison, WI). Reverse transcription was performed following the manufacturer‐supplied protocol in a 20 *μ*L reaction mixture containing oligo(dT) primer. PCR products were generated using an LA Taq polymerase kit (Takara, Japan) with 2 *μ*L of cDNA applied to PCR using specific primers. PCR product separation was achieved using a 1% agarose gel, followed by visualization under ultraviolet light subsequent to ethidium bromide staining. PCR product band intensities were determined using Raytest TINA densitometry software version 2.09c (Straubenhardt, Germany).

### Protein extraction and western blot analysis

Cells washed with PBS were lysed using radioimmunoprecipitation assay buffer (Pierce, Rockford, IL) that was supplemented with Roche complete protease inhibitor cocktail tablets (Nutley, NJ). Protein extracts were collected via centrifugation at 14,000*g* for 20 min and protein concentrations were determined with an RC/DC Bio‐Rad assay kit (Bio‐Rad, Hercules, CA) following the manufacturer‐supplied protocol. Protein extracts were separated via sodium dodecyl sulfate–polyacrylamide gel electrophoresis and transferred to polyvinylidene fluoride membranes (PALL Corp., Port Washington, NY). The membranes were preincubated with blocking solution (3% (w/v) skim milk in TBS containing 0.1% Tween‐20) for 1 h, incubated with anti‐VEGF‐A, anti‐VEGF‐C, anti‐VEGFR‐1, anti‐VEGFR‐2, anti‐VEGFR‐3 (1:2000 dilution in blocking solution; Santa Cruz Biotechnology, Inc., Santa Cruz, CA), or anti‐*β*‐actin (1:5000 dilution in blocking solution; Santa Cruz Biotechnology, Inc.) antibodies overnight at 4°C, then probed with peroxidase‐conjugated anti‐mouse IgG, anti‐goat IgG, or anti‐rabbit IgG (1:5000 dilution in blocking solution; Santa Cruz Biotechnology, Inc.). Protein bands were detected using enhanced chemiluminescent western blotting detection reagents (GE Healthcare, Piscataway, NJ).

### In vivo tumor studies

C3H/HeN 5‐week‐old male mice purchased from Orient Bio Inc. (Seongnam, Korea) were provided with water and food ad libitum, and they were quarantined in a specific pathogen‐free facility accredited by the Kyung Hee University Institutional Animal Care and Use Committee with a 12‐h light/dark photoperiod. Animal care and all experimental procedures followed the Kyung Hee University guidelines regarding care and use of laboratory animals.

An orthotropic oral SCC animal model was established by the injection of 5 × 10^5^ SCC‐VII cells in 50 *μ*L PBS into the anterior buccal mucosa of C3H/HeN mice. Tumors grew for 8 days to form visible masses. Mice were then randomly divided into two groups (*n* = 8) and treated with a daily peritumoral injection of either recombinant canstatin at 5 mg/kg/day in PBS, or with a PBS for 13 days. Mice were sacrificed at 28 days posttumor inoculation and tumors were weighed after excision. A caliper was used to determine tumor lengths and widths. Tumor volumes were calculated using the standard formula: [length × width squared × 0.5] 18.

### Matrigel plug assay

Matrigel aliquots of 0.3 mL containing 500 ng/mL rhVEGF‐A and 40 *μ*g/mL recombinant canstatin were injected bilaterally into the flank areas of 5‐week‐old female BALB/c mice (Orient Bio Inc.). Seven days postinjection, excised Matrigel plugs were fixed in 4% (v/v) paraformaldehyde prior to immunohistochemical analysis.

### Immunohistochemistry

Primary tumors were removed and fixed overnight in 10% (v/v) neutral buffered formalin. Tumors and Matrigel plugs were embedded in paraffin. Paraffin‐embedded tumors and Matrigel plugs were sectioned to 5 *μ*m thickness, deparaffinized via immersion in xylene, and dehydrated in a graded series of ethanol followed by washing in distilled water. Thereafter, the sections were boiled in 10 mmol/L sodium citrate buffer (pH 6.0) for 10 min, cooled to room temperature, and then incubated with methanol containing 1% (v/v) hydrogen peroxide for 10 min to inhibit endogenous peroxidase activity. The sections were blocked with blocking solution (10% (v/v) normal goat serum, Dako, Glostrup, Denmark) for 1 h and incubated overnight with primary antibodies (anti‐CD31, anti‐LYVE‐1 [Abcam, Cambridge, UK], anti‐VEGF‐A, anti‐VEGF‐C, anti‐VEGFR‐1, anti‐VEGFR‐2, or anti‐VEGFR‐3) diluted with blocking solution. The sections were probed using peroxidase‐conjugated secondary antibodies, followed by incubation in a peroxidase substrate solution (Vector Laboratories, Burlingame, CA) for development of appropriate stain intensity. Subsequent to counterstaining with Harris hematoxylin, all sections were subjected to examination using a BX21‐inverted microscope (Olympus, Japan). For the analysis of immunohistochemical signals in the tumor specimens and Matrigel plugs, digital images of all sections were captured under 400× objective magnification and analyzed using the Image J program.

### Statistical analysis

All data are represented as mean ± standard deviation (SD) or standard error (SE). Student's *t*‐test was used for comparison between nontreated control and treated data groups (*P <* *0.05, **P <* *0.01, ***P <* *0.001).

## Results

### Effects of recombinant canstatin on tumor growth and lung metastasis in an orthotropic oral SCC‐VII animal model

**Figure 1:**
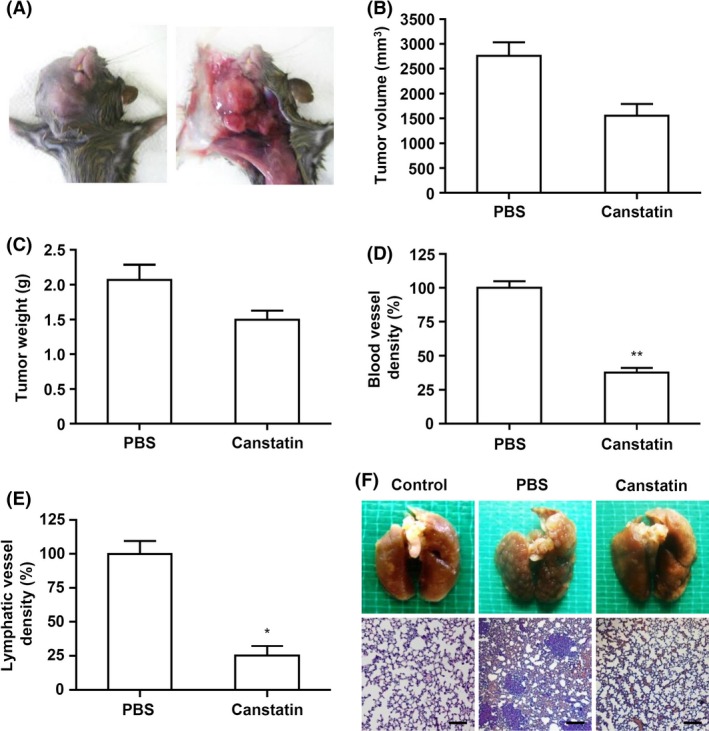
Effects of recombinant canstatin on the final volume and weight of squamous cell carcinoma (SCC)‐VII‐induced tumors, blood and lymphatic vessel densities, and lung metastasis. SCC‐VII cells (5 × 10^5^ cells in 50 *μ*L PBS) were injected into the anterior buccal mucosa of C3H/HeN mice. Eight days after cell inoculation, mice were treated daily with a peritumoral injection of either recombinant canstatin (5 mg/kg/day in PBS) or PBS (control) for 13 days. Twenty‐eight days after cell inoculation, tumors in mice were photographed (A). All mice were sacrificed, and their tumor volumes (B) and tumor weights (C) were measured. The number of blood and lymphatic vessels in tumor sections of each group was determined using immunohistochemical analysis with anti‐CD31 and anti‐LYVE‐1 antibodies, respectively. The number of blood vessels per mm^2^ is expressed as the blood vessel density (D). The number of lymphatic vessels per mm^2^ is expressed as the lymphatic vessel density (E). The blood and lymphatic vessel densities of the control group were established as 100%. Data are presented as mean ± SE (*P <* *0.05, **P <* *0.01). (F) The lungs were also removed from all mice and photographed. Lung tissues were fixed in 10% (v/v) neutral buffered formalin overnight and stained with hematoxylin and eosin solution. The control is a lung from a normal mouse that was not inoculated with SCC‐VII cells. Scale bar = 100 *μ*m.

Antitumor effects of recombinant canstatin were investigated using a C3H/HeN mouse‐based orthotropic oral SCC‐VII animal model (Fig. 1A). In control group mice treated with PBS, rapid tumor growth produced an average volume of 2760.4 ± 270.4 mm^3^ (mean ± SE) 28 days after SCC‐VII cell inoculation. The primary tumor volume in 5 mg/kg/day recombinant canstatin‐treated mice was 1552.5 ± 236.4 mm^3^, which is equivalent to 56.2% of control volume (Fig. 1B). The mean tumor weight for 5 mg/kg/day recombinant canstatin‐treated mice was reduced to 72.8% of the value for control group mice (Fig. 1C).

Tumor capillaries were identified using immunohistochemical CD31 staining for blood vessels and hyaluronan receptor‐1 (LYVE‐1) staining for lymphatic vessels. Control group mice tumor specimens exhibited an average blood vessel density value of 8.67 ± 0.42 per mm^2^ (mean ± SE). In contrast, the blood vessel density of the 5 mg/kg/day recombinant canstatin‐treated group mice was 3.25 ± 0.3 per mm^2^, which is equivalent to 37.5% of the density value for control group mice (Fig. 1D). Tumors were also analyzed for a lymphangiogenesis marker based on LYVE‐1 immunohistochemical staining. Tumors of control group mice exhibited an average lymphatic vessel density value of 5.83 ± 0.77 per mm^2^ (mean ± SE). The lymphatic vessel density value of the 5 mg/kg/day recombinant canstatin‐treated group mice was 1.47 ± 0.39 per mm^2^, which is equivalent to 25.2% of the density value for control group mice (Fig. 1E). The occurrence of lung metastasis was also investigated at 5 weeks posttumor inoculation. Metastases were detected in most SCC‐VII‐inoculated and PBS‐treated mice (Fig. 1F). Lung metastasis was reduced to 37% (three of eight mice) in recombinant canstatin‐treated group mice. Lung metastasis via spreading of SCC‐VII cells was confirmed based on histopathological analysis using hematoxylin and eosin staining. Taken together, these results indicate that recombinant canstatin inhibits primary tumor growth in an orthotropic oral SCC‐VII animal model. In addition, recombinant canstatin inhibited lung metastasis caused by the spreading of SCC‐VII‐induced tumors.

### Effects of recombinant canstatin on the expression of VEGF family proteins in CoCl_2_‐treated SCC‐VII cells

**Figure 2:**
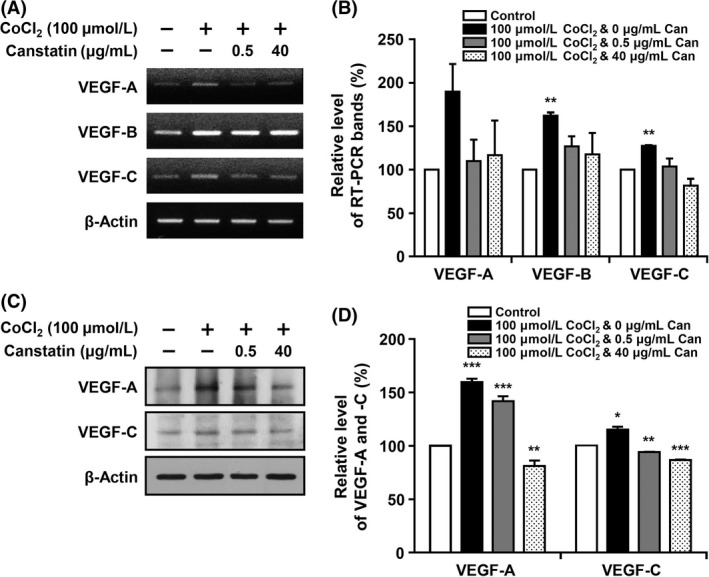
Effects of recombinant canstatin on the expression of vascular endothelial growth factor (VEGF) family proteins in CoCl_2_‐treated squamous cell carcinoma (SCC)‐VII cells. (A) SCC‐VII cells were treated with different concentrations of recombinant canstatin (0, 0.5, 40 *μ*g/mL) in the presence of 100 *μ*mol/L CoCl_2_, and incubated for 24 h. cDNAs were generated from DNase I‐treated total RNA, and PCR was performed with specific primers for vascular endothelial growth factor (VEGF)‐A, ‐B, ‐C, and *β*‐actin. (B) The PCR products from three independent experiments in (A) were quantified and are represented as a bar diagram. The transcript levels of VEGF‐A, ‐B, and ‐C mRNA in the control (recombinant canstatin‐ and CoCl_2_‐untreated cells) were established as 100%. (C) Protein levels of VEGF‐A and ‐C in the intracellular fraction were determined using western blot analysis with anti‐VEGF‐A and anti‐VEGF‐C antibodies. (D) The amounts of VEGF‐A and ‐C obtained in three independent experiments of (C) were quantified and are represented as a bar diagram. The levels of VEGF‐A and ‐C in the control were established as 100%. Data are presented as mean ± SD of three independent experiments (*P <* *0.05, **P <* *0.01, ***P <* *0.001).

For analyzing the effects of recombinant canstatin on VEGF family gene expression, total RNA was prepared from CoCl_2_‐treated SCC‐VII cells in the presence or absence of recombinant canstatin. RT‐PCR‐based measurements with *β*‐actin as an internal control indicated that the VEGF‐A mRNA transcript level increased 89.7% in CoCl_2_‐treated cells (Fig. 2A and 2B). Increased expression of the VEGF‐A transcript due to CoCl_2_ was reduced by 89.3% in 0.5 *μ*g/mL and by 81.4% in 40 *μ*g/mL recombinant canstatin‐treated cells. Treatment with CoCl_2_ also increased levels of VEGF‐B and ‐C mRNAs by 61.9% and 27.3%, respectively. Use of 0.5 or 40 *μ*g/mL recombinant canstatin decreased expression of VEGF‐B transcripts by 57% and 71.9%, respectively. VEGF‐C mRNA transcript levels decreased by 87% in both CoCl_2_‐treated and 0.5 *μ*g/mL recombinant canstatin‐treated cells. The presence of 40 *μ*g/mL recombinant canstatin further reduced the expression level of the VEGF‐C mRNA transcript below the level of the CoCl_2_‐untreated control. Expression of the VEGF‐A protein in CoCl_2_‐treated cells was further confirmed using western blot analysis (Fig. 2C and 2D). The VEGF‐A protein level in CoCl_2_‐treated cells increased by 59.6% compared to the level in CoCl_2_‐untreated cells. The level of VEGF‐A protein in CoCl_2_‐treated and 0.5 *μ*g/mL recombinant canstatin‐treated cells was reduced by 30.1% compared to the level in CoCl_2_‐treated cells. The presence of 40 *μ*g/mL recombinant canstatin further reduced the protein expression level of VEGF‐A below the level of the CoCl_2_‐untreated control. The level of the VEGF‐C protein increased in CoCl_2_‐treated cells by 15.2% compared to the level in CoCl_2_‐untreated cells. Use of both 0.5 and 40 *μ*g/mL recombinant canstatin reduced the expression level of the VEGF‐C protein below the level of the CoCl_2_‐untreated control. These results indicate that VEGF‐A is the most significantly elevated VEGF family protein in SCC‐VII cells in response to hypoxia, and recombinant canstatin decreases the expression level of VEGF‐A.

### Effects of recombinant canstatin on lymphatic vessel formation in an in vivo Matrigel plug

**Figure 3:**
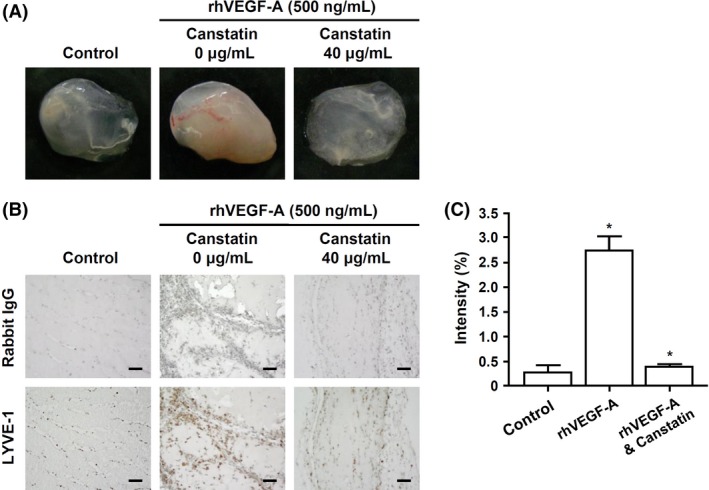
An in vivo Matrigel plug assay. (A) Matrigel aliquots containing 500 ng/mL rhVEGF‐A and/or 40 *μ*g/mL recombinant canstatin were injected bilaterally into the flank areas of BALB/c mice. Seven days after injection, Matrigel plugs were excised and imaged using a digital camera. (B) The lymphatic vessel densities in Matrigel plug sections were determined using immunohistochemical analysis with anti‐LYVE‐1 antibody. All Matrigel sections were digitalized and images were captured under 400× objective magnification. Scale bar = 100 *μ*m. (C) Immunohistochemical intensities of LYVE‐1 from captured images were analyzed using the Image J program and are represented as a bar diagram. Data are presented as mean ± SE (*P <* *0.05).

To determine the effect of recombinant canstatin on rhVEGF‐A‐induced lymphangiogenesis in vivo, a Matrigel plug assay was performed using a BALB/c mice‐based animal model. Matrigel plugs containing 500 ng/mL rhVEGF‐A were treated with 40 *μ*g/mL recombinant canstatin or PBS (control), injected bilaterally into the inguinal areas, and subsequently removed 14 days postimplantation, followed by photography and immunohistological examination. rhVEGF‐A‐treated Matrigel plugs contained red‐colored capillary vessels, indicating formation of functional vasculature inside the Matrigel (Fig. 3A). In contrast, formation of capillary vessels was inhibited in Matrigel plugs treated with both rhVEGF‐A and recombinant canstatin. For immunohistological analysis, Matrigel plug sections were stained with hematoxylin and anti‐LYVE‐1 antibody (Fig. 3B). Lymphatic vessel density indicated by anti‐LYVE‐1 staining was significantly increased in rhVEGF‐A‐treated Matrigel plugs. The staining intensity of lymphatic vessels in recombinant canstatin‐treated Matrigel plugs decreased by 86% compared with rhVEGF‐A‐treated Matrigel plugs (Fig. 3C). These results indicate that rhVEGF‐A enhances in vivo lymphatic vessel formation, and that recombinant canstatin reduces the lymphatic vessel formation that is induced by rhVEGF‐A.

### Effects of recombinant canstatin on the expression of VEGFR‐1 and ‐2 in rhVEGF‐A‐treated HLMECs

**Figure 4:**
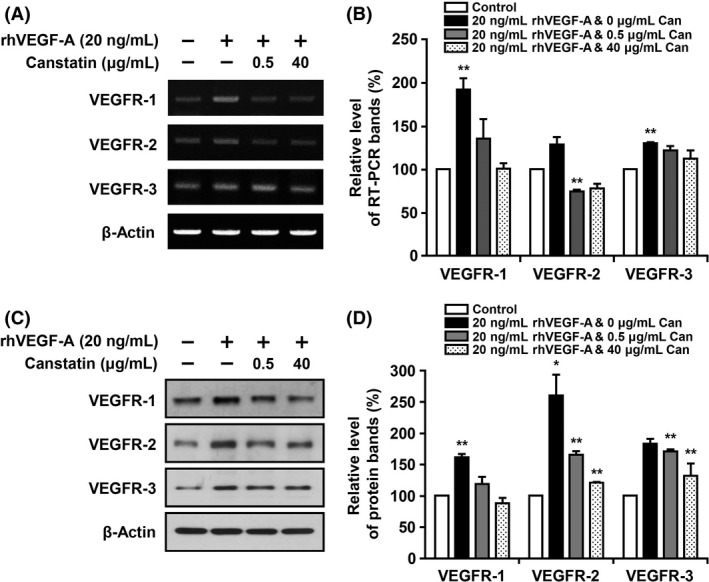
Effects of recombinant canstatin on the expression of vascular endothelial growth factor receptors (VEGFR)‐1, ‐2, and ‐3 in rh vascular endothelial growth factor (VEGF)‐A‐treated HLMECs. (A) HLMECs were treated with different concentrations (0, 0.5, 40 *μ*g/mL) of recombinant canstatin in the presence of 20 ng/mL of rhVEGF‐A. DNase I‐treated total RNAs were prepared 24 h after incubation, and RT‐PCR was performed. (B) The amounts of PCR product obtained in three independent experiments of (A) were quantified and are represented as a bar diagram. The levels of the VEGFR‐1, ‐2, and ‐3 mRNA transcripts in recombinant canstatin‐ and rhVEGF‐A‐untreated cells were established as 100%. (C) Levels of VEGFR‐1, ‐2, and ‐3 proteins in the intracellular fractions were determined using western blot analysis. (D) The amounts of VEGFR‐1, ‐2, and ‐3 obtained in three independent experiments of (C) were quantified and are represented as a bar diagram. The levels of VEGFR‐1, ‐2, and ‐3 in recombinant canstatin‐ and rhVEGF‐A‐untreated cells were established as 100%. Data are presented as mean ± SD of three independent experiments (*P <* *0.05, **P <* *0.01).

The mRNA transcript levels of the VEGF family protein receptors (VEGFR‐1, ‐2, and ‐3) in rhVEGF‐A‐treated and recombinant canstatin‐treated HLMECs were detected using RT‐PCR (Fig. 4A and 4B). Total RNA was prepared from HLMECs treated with 20 ng/mL rhVEGF‐A in the presence or absence of recombinant canstatin (0.5 or 40 *μ*g/mL). RT‐PCR measurements with *β*‐actin as an internal control indicated that treatment with rhVEGF‐A increased VEGFR‐1, ‐2, and ‐3 mRNA levels by 91.7%, 28.3%, and 29.7%, respectively, compared to those in rhVEGF‐A‐untreated controls. Recombinant canstatin at 0.5 or 40 *μ*g/mL reduced the expression levels of VEGFR‐1 mRNA by 61.8% and 99.5%, respectively, compared to that in rhVEGF‐A‐treated cells. Treatment with 0.5 or 40 *μ*g/mL recombinant canstatin reduced the expression level of VEGFR‐2 to less than that of the rhVEGF‐A‐untreated control. The presence of 0.5 or 40 *μ*g/mL recombinant canstatin reduced the expression of VEGFR‐3 mRNA by 27.6% and 59.9%, respectively, compared to that in rhVEGF‐A‐treated cells.

VEGFR‐1, ‐2, and ‐3 protein expression levels were also examined in HLMECs treated with rhVEGF‐A and recombinant canstatin via western blot analysis (Fig. 4C and 4D). Treatment with rhVEGF‐A increased the levels of VEGFR‐1, ‐2, and ‐3 proteins by 61.1%, 159.9%, and 82.9%, respectively, compared to those in the rhVEGF‐A‐untreated control. Treatment with 0.5 *μ*g/mL recombinant canstatin decreased the VEGFR‐1 expression level by 69.4% compared to that in rhVEGF‐A‐treated cells. Treatment with 40 *μ*g/mL recombinant canstatin decreased the VEGFR‐1 expression level to less than that of the rhVEGF‐A‐untreated control. Treatment with 0.5 or 40 *μ*g/mL recombinant canstatin decreased the expression of VEGFR‐2 by 59.3% and 87%, respectively, compared to that in rhVEGF‐A‐treated cells. The expression of VEGFR‐3 was reduced by 15.2% and 61.8%, respectively, in HLMECs treated with 0.5 or 40 *μ*g/mL recombinant canstatin. These results indicate that recombinant canstatin inhibits the expression of VEGFR‐1, ‐2, and ‐3 induced by rhVEGF‐A in HLMECs.

### Effects of recombinant canstatin on the expression of VEGF‐A, VEGFR‐1, and VEGFR‐2 in SCC‐VII‐induced tumors

**Figure 5:**
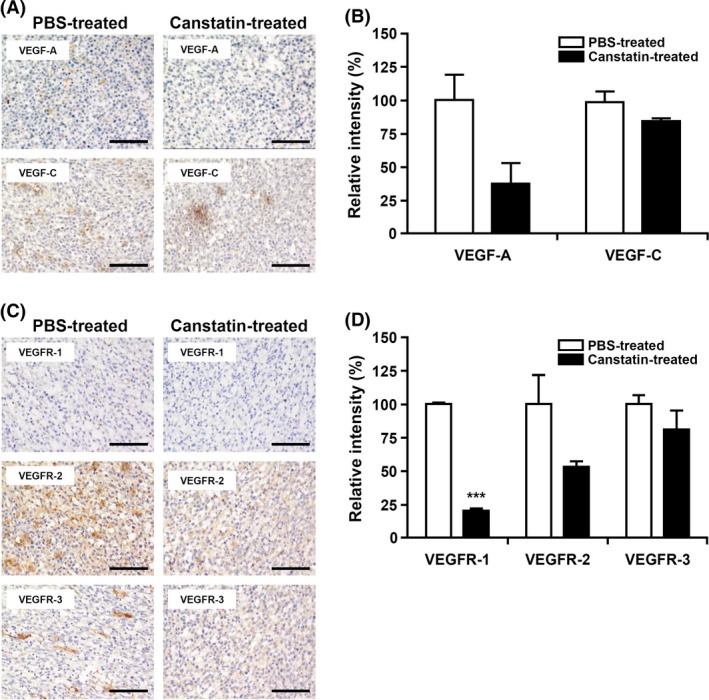
Effects of recombinant canstatin on the expression of vascular endothelial growth factor (VEGF)‐A, VEGF‐C, vascular endothelial growth factor receptors (VEGFR)‐1, VEGFR‐2, and VEGFR‐3 in squamous cell carcinoma (SCC)‐VII‐induced tumors. (A, C) The presence of VEGF‐A, VEGF‐C (A), VEGFR‐1, VEGFR‐2, and VEGFR‐3 (C) in the sections of PBS‐treated or 5 mg/kg/day recombinant canstatin‐treated SCC‐VII‐induced tumors was determined using immunohistochemical analysis. All tumor sections were digitized and images were captured under a 400× objective magnification. Scale bar = 100 *μ*m. (B, D) Immunohistochemical intensities of VEGF‐A, VEGF‐C (B), VEGFR‐1, VEGFR‐2, and VEGFR‐3 (D) from captured images were analyzed using the Image J program and are represented as bar diagrams. The intensities of the VEGF‐A, VEGF‐C, VEGFR‐1, VEGFR‐2, and VEGFR‐3 staining of the PBS‐treated control were established as 100%. Data are presented as mean ± SD of three independent experiments (***P <* *0.001).

The effects of recombinant canstatin on the expression of VEGF‐A and VEGF‐C in SCC‐VII‐induced tumors were evaluated using immunohistochemical analysis. Tumor sections were prepared from primary tumor specimens of PBS‐ or recombinant canstatin‐treated mice and subjected to immunohistochemical analysis to estimate the expression levels of VEGF‐A and VEGF‐C. The expression of VEGF‐A and VEGF‐C decreased in recombinant canstatin‐treated tumors by 62.7% and 14.5%, respectively, compared to those of PBS‐treated controls (Fig. 5A and 5B). The expression of VEGFR‐1, ‐2, and ‐3 was also determined using immunohistochemical analysis in SCCV‐II oral squamous cell carcinoma‐induced tumors. Treatment with recombinant canstatin caused decreases in expression levels of VEGFR‐1, ‐2, and ‐3 of 79.9%, 47.1%, and 19.5%, respectively, in recombinant canstatin‐treated tumors, compared to those of PBS‐treated tumors (Fig. 5C and 5D). These results indicate that recombinant canstatin significantly inhibits the expression of VEGF‐A, VEGFR‐1, and VEGFR‐2 in SCC‐VII‐induced tumors.

### Effects of recombinant canstatin on the activation of VEGFR‐1 and ‐2 in rhVEGF‐A‐treated HLMECs

**Figure 6:**
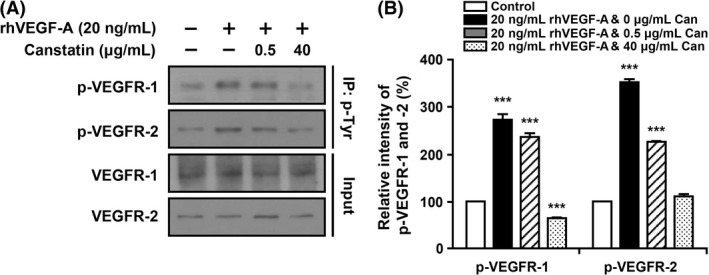
Effect of recombinant canstatin on the phosphorylation of vascular endothelial growth factor receptors (VEGFR)‐1 and ‐2 in rh vascular endothelial growth factor (VEGF)‐A‐treated HLMECs. (A) HLMECs were treated with different concentrations of recombinant canstatin (0, 0.5, 40 *μ*g/mL) in the presence of 20 ng/mL of rhVEGF‐A. Cell lysates were immunoprecipitated with anti‐phospho‐Tyr (anti‐p‐Tyr) antibody. The presence of p‐VEGFR‐1 and p‐VEGFR‐2 in immunoprecipitates was detected via western blot analysis using anti‐VEGFR‐1 and anti‐VEGFR‐2 antibodies. (B) The amounts of p‐VEGFR‐1 and p‐VEGFR‐2 determined in three independent experiments of (A) were quantified and are represented as a bar diagram. The levels of p‐VEGFR‐1 and p‐VEGFR‐2 in recombinant canstatin‐ and rhVEGF‐A‐untreated cells were established as 100%. Data are presented as mean ± SD of three independent experiments (*P <* *0.05).

To investigate the effect of recombinant canstatin on the activation of VEGFR‐1 and ‐2 in rhVEGF‐A‐treated HLMECs, the phosphorylation levels of VEGFR‐1 and ‐2 were determined based on immunoprecipitation using an anti‐p‐Tyr antibody, followed by western blot analysis. Cell lysates prepared from HLMECs treated with 20 ng/mL rhVEGF‐A in the presence or absence of recombinant canstatin (0.5 or 40 *μ*g/mL) were immunoprecipitated with anti‐p‐Tyr. The presence of phospho‐VEGFR‐1 (p‐VEGFR‐1) and ‐2 in immunoprecipitates was determined via western blot analysis using anti‐VEGFR‐1 and anti‐VEGFR‐2 (Fig. 6A). The level of p‐VEGFR‐1 and ‐2 increased by 172.5% and 251.7%, respectively, in rhVEGF‐A‐treated cells compared to those in the rhVEGF‐A‐untreated control. Treatment with 0.5 *μ*g/mL recombinant canstatin decreased p‐VEGFR‐1 level by 13.4% compared to that in rhVEGF‐A‐treated HLMECs. The level of p‐VEGFR‐1 in 40 *μ*g/mL recombinant canstatin‐treated cells was less than that of the rhVEGF‐A‐untreated control. Treatment with 0.5 or 40 *μ*g/mL recombinant canstatin decreased p‐VEGFR‐2 levels by 50.1% and 95.9%, respectively, compared to that of rhVEGF‐A‐treated cells. These results indicate that recombinant canstatin inhibits the activation of VEGFR‐1 and ‐2 induced by VEGF‐A in HLMECs.

## Discussion

Oral cancer is the most commonly occurring cancer in head and neck areas, with SCC being the most common single type. This study investigated the antitumor activities of recombinant canstatin in an orthotropic oral SCC‐VII animal model using C3H/HeN mice. A dosage of 5 mg/kg/day of recombinant canstatin was used in an in vivo experiment based on our previous experiment using an orthotopic AT‐84 oral squamous cell carcinoma model in which a peritumoral injection of recombinant canstatin at a dosage of 5 mg/kg/day successfully inhibited tumor growth induced by AT‐84 15. Recombinant canstatin resulted in reduced tumor sizes and weights in the animal model (Fig. 1A and 1B), as was similarly observed in a heterotopic CT‐26 colon carcinoma animal model 16. Lymphatic and blood vessel densities significantly decreased in recombinant canstatin‐treated tumors (Fig. 1D and 1E). These results indicate that recombinant canstatin exhibits antiangiogenic and antilymphangiogenic effects in an SCC‐VII animal model, resulting in the inhibition of tumor growth. Moreover, recombinant canstatin significantly suppressed lung metastasis of oral SCC through lymph node metastasis (Fig. 1F). Generally, oral cancer initially metastasizes to regional lymph nodes via the lymphatic system and lungs, bones, and liver are the most common sites of distant metastases. Tumor‐induced lymphangiogenesis plays an important role in the metastatic spread of oral cancer. Lymphatic spread of HNSCC is important because malignant cancer cells preferentially metastasize to many lymph nodes in the cervical region. Lymph node spread is the most powerful prognostic factor for the survival of HNSCC patients. However, details of molecular mechanisms and processes of lymphatic metastasis have not been well understood. Lymphangiogenesis has been closely related to lymphatic metastasis and the lymphatic system provides easy movement of cancer cells 19, 20, 21, 22. Tumor‐induced lymphangiogenesis is facilitated by lymphangiogenic growth factors, including the VEGF family, the angiopoietin family, FGF, PDGFs, and other factors 5, 23. Previously, the expression of lymphangiogenic growth factors was examined in CoCl_2_‐treated SCC‐VII cells. CoCl_2_, a hypoxia mimetic agent, allows for the preferential growth of hypoxic carcinoma cells over control cells, resulting in a higher cell number 24. Treatment with CoCl_2_ increased the mRNA transcript levels of VEGF‐A, ‐B, ‐C, insulin‐like growth factor, hepatocyte growth factor, and FGF‐1 (data not shown). CoCl_2_ highly increased the expression of VEGF‐A in SCC‐VII cells, but this increase was reduced by recombinant canstatin (Fig. 2). However, recombinant canstatin did not affect the transcript expression of VEGF‐B, ‐C, or other growth factors (data not shown). In response to hypoxia, hypoxia‐inducible factor‐1 (HIF‐1), a heterodimeric transcription factor consisting of two subunits, HIF‐1*α* and HIF‐1*β*, upregulates the expression of VEGF family genes 25. Mitochondria plays a key role in the transcriptional regulation of VEGF by HIF‐1 in response to hypoxia 26, 27. Canstatin reduces mitochondrial membrane potential and induces mitochondrial damage 14, 28. Taken together, we speculate that the reduction in mitochondrial membrane potential by recombinant canstatin might suppress the transcriptional activation of VEGF by HIF‐1 in response to hypoxia.

VEGF‐A is a predominant tumor angiogenesis factor that plays a principle role in tumor progression and angiogenesis 29. However, several studies have revealed that VEGF‐A can promote the migration and proliferation of LECs 30, 31. Adenoviral murine VEGF‐A injection stimulates in vivo lymphangiogenesis in the skin of the mouse ear 32. Targeted VEGF‐A overexpression induces tumor lymphangiogenesis in cutaneous SCC and promotes tumor metastasis to sentinel lymph nodes 11. In our preliminary experiment, VEGF‐A dose‐dependently enhanced the proliferation of HLMECs (data not shown). Recombinant canstatin reduced the proliferation of HLMECs induced by VEGF‐A (Fig. S1). Recombinant canstatin alone dose‐dependently inhibited the proliferation of LECs 16. To evaluate the antilymphangiogenic effects of recombinant canstatin, concentrations of 0.5 and 40 *μ*g/mL were used for mild and moderate levels of cell proliferation inhibition 16. Both tube formation and migration induced by VEGF‐A in HLMECs were inhibited by recombinant canstatin, even under conditions that did not induce the death of HLMECs (Fig. S2). Antilymphangiogenic activities of recombinant canstatin were investigated via an in vivo Matrigel plug assay using BALB/c mice. VEGF‐A stimulated in vivo formation of lymphatic vessels within Matrigel plugs (Fig. 3). The density of lymphatic vessels significantly decreased in Matrigel plugs treated with recombinant canstatin based on anti‐LYVE‐1 antibody staining. These results strongly indicate that VEGF‐A is one of the lymphangiogenic factors produced by SCC‐VII cells under hypoxic conditions, and that recombinant canstatin decreases the formation of lymphatic vessels induced by VEGF‐A.

VEGF‐A binds to the cell surface receptors VEGFR‐1 and ‐2, tyrosine kinases expressed by endothelial cells, and induces the growth of new blood vessels from preexisting vessels 29. VEGFR‐1 and ‐2 receptors act through different pathways to contribute to endothelial cell proliferation, migration, and the formation of tubular structures 29, 33. VEGFR‐2 is the predominant mediator of VEGF‐A‐related responses in vascular endothelial cells, whereas VEGFR‐1 exhibits a higher affinity for VEGF‐A with a weaker tyrosine kinase activity. VEGF‐A stimulates the activation of VEGFR‐2 in LECs and promotes tissue repair‐associated lymphatic vessel formation and lymphangiogenesis 31, 34. Recombinant canstatin suppressed the expression of VEGFR‐1 and ‐2 that was induced by VEGF‐A in HLMECs (Fig. 4). VEGF‐A stimulates the expression of VEGFR‐2 in HUVECs, which is mediated by the VEGF‐A‐stimulated activation of extracellular signaling‐regulated kinase 1/2 (ERK1/2) and phosphatidylinositol 3‐kinase (PI3K)/Akt signaling 35. Recombinant canstatin decreased the phosphorylation of ERK1/2, PI3K, and Akt stimulated by VEGF‐A in HLMECs (data not shown). This suggests that recombinant canstatin inhibits VEGF‐A‐stimulated activation signals of PI3K, AKT, and ERK1/2 and suppresses the expression of VEGFR‐1 and ‐2 in HLMECs. The expression of VEGF‐A and VEGFR‐1 and ‐2 was reduced in SCC‐VII‐induced tumors by treatment with recombinant canstatin (Fig. 5). These results indicate that recombinant canstatin reduces the tumor growth induced by oral SCC, which is mediated by a decrease in levels of VEGF‐A and VEGFR‐1 and ‐2. Recombinant canstatin decreased the phosphorylation of VEGFR‐1 and ‐2 induced by VEGF‐A (Fig. 6). Recombinant canstatin also inhibited the activation of signaling factors (focal adhesion kinase, ERK1/2, Akt, PI3K, c‐Jun N‐terminal kinases, and p38) involved in VEGF‐A and VEGFR‐1/VEGFR‐2 signaling (data not shown). These results suggest that the antilymphangiogenic effects of recombinant canstatin are probably mediated via the suppression of VEGF‐A/VEGFR‐1 and ‐2 signaling in HLMECs.

Integrin, a heterodimeric transmembrane glycoprotein constituted of *α* and *β* subunits, is important for the regulation of lymphangiogenesis and angiogenesis during normal development, and plays a key role in several diseases 36. Integrin mediation of cell–cell and cell–endothelial cell matrix connections controls the adhesion, migration, and survival of vascular and lymphatic endothelial cells 37, 38. Integrins *α*1*β*1 and *α*2*β*1 are implicated in lymphangiogenesis as a response to VEGF‐A; their expression is enhanced by VEGF‐A in LECs, promoting the capacity of LECs to form cords and migrate 31. Integrin *α*4*β*1 that is expressed on tumor and growth factor‐induced lymphatic endothelium regulates the adhesion, migration, invasion, and survival of LECs 39. Integrin *α*v*β*3 that is mainly expressed in proliferating endothelial cells mediates capillary formation. Integrin *α*v*β*3 interacts with VEGFR‐2 and regulates cellular activities involved in angiogenesis, including endothelial cell migration, survival, and tube formation, as well as hematopoietic cell functions within the vasculature 40. Canstatin interacts with integrins *α*v*β*3 and *α*v*β*5, mediating a mitochondrial apoptotic process in endothelial and tumor cells 28. In our experiments, treatment with anti‐*α*v*β*3 antibody blocked the phosphorylation of VEGFR‐1 and ‐2 in VEGF‐A‐stimulated HLMECs (Fig. S3). The presence of recombinant canstatin augmented the inhibitory effect of anti‐*α*v*β*3. These results suggest that integrin *α*v*β*3 is probably involved in lymphangiogenesis induced by VEGF‐A/VEGFR‐1 and ‐2 signaling, and that the antilymphangiogenic effects of recombinant canstatin may be caused by the suppression of integrin *α*v*β*3/VEGFR‐1 and/or ‐2 signaling stimulated by VEGF‐A.

In conclusion, VEGF‐A is one of the lymphangiogenic factors expressed by SCC‐VII cells in hypoxic conditions. Recombinant canstatin reduced the expression of VEGF‐A in CoCl_2_‐treated SCC‐VII cells. VEGF‐A promoted in vivo formation of lymphatic vessels in a Matrigel plug, which was inhibited by the treatment with recombinant canstatin. Recombinant canstatin suppressed the expression and activation of VEGFR‐1 and ‐2 induced by VEGF‐A in HLMECs and SCC‐VII‐induced tumors. These findings indicate that recombinant canstatin has antitumoral and antilymphangiogenic activities against oral SCC cells, inhibiting lymphangiogenic signaling induced by VEGF‐A. Recombinant canstatin probably inhibits lymphangiogenesis via suppression of integrin *α*v*β*3/VEGFR‐1 and/or ‐2 signaling induced by VEGF‐A. Recombinant canstatin reduced the final volumes and weights of oral SCC‐induced tumors, and the degree of lung metastasis of oral SCC via lymph node metastasis. Our results suggest that recombinant canstatin can be a valuable therapeutic agent to treat oral SCC‐induced tumors and prevent metastatic spread of oral SCC.

## Conflict of Interest

None declared.

## Supporting information


**Figure S1.** Recombinant canstatin reduced the proliferation of HLMECs stimulated with rhVEGF‐A. HLMECs (5 × 104 cells/well in EBM‐2 containing 2% FBS) were added to each well of gelatinized 24‐well plates and treated with different concentrations (0, 0.1, 0.5, 1, 5, 10, 20, 40, and 60 μg/ml) of recombinant canstatin in the presence of 20 ng/ml of rhVEGF‐A for 48 h.
**Figure S2.** Recombinant canstatin reduced the tube formation and migration of HLMECs stimulated with rhVEGF‐A. (A) HLMECs in EBM‐2 containing 2% FBS were added to Matrigel‐precoated 48‐well plates and treated with different concentrations (0, 0.5, and 40 μg/ml) of recombinant canstatin in the presence of 20 ng/ml of rhVEGF‐A.
**Figure S3.** The phosphorylation of VEGFR‐1 and ‐2 by VEGF‐A is reduced by the presence of anti‐αvβ3 and recombinant canstatin.
