# Supplementary material for: Recombinant canstatin inhibits VEGF‐A‐induced lymphangiogenesis and metastasis in an oral squamous cell carcinoma SCC‐VII animal model
Source: Cancer Med. 2016 Sep 20;5(10):2977–88. doi: 10.1002/cam4.866 (PMC5083751; doi:10.1002/cam4.866)
Supplement: Supplementary file 1 — Figure S1. Recombinant canstatin reduced the proliferation of HLMECs stimulated with rhVEGF‐A. HLMECs (5 × 10^4^ cells/well in EBM‐2 containing 2% FBS) were added to each well of gelatinized 24‐well plates and treated with different concentrations (0, 0.1, 0.5, 1, 5, 10, 20, 40, and 60 μg/ml) of recombinant canstatin in the presence of 20 ng/ml of rhVEGF‐A for 48 h. Figure S2. Recombinant canstatin reduced the tube formation and migration of HLMECs stimulated with rhVEGF‐A. (A) HLMECs in EBM‐2 containing 2% FBS were added to Matrigel‐precoated 48‐well plates and treated with different concentrations (0, 0.5, and 40 μg/ml) of recombinant canstatin in the presence of 20 ng/ml of rhVEGF‐A. Figure S3. The phosphorylation of VEGFR‐1 and ‐2 by VEGF‐A is reduced by the presence of anti‐αvβ3 and recombinant canstatin. [file CAM4-5-2977-s001.pdf]

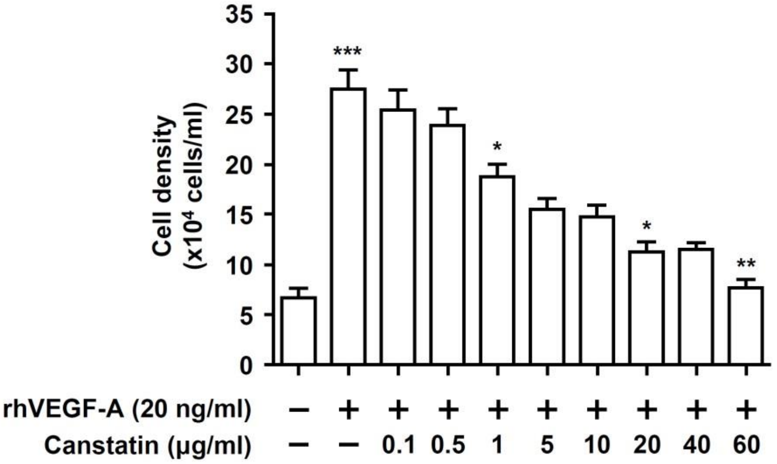

Supporting Information Fig. 1. Recombinant canstatin reduced the proliferation of HLMECs stimulated with rhVEGF-A. HLMECs (5×10^4 cells/well in EBM-2 containing 2% FBS) were added to each well of gelatinized 24-well plates and treated with different concentrations (0, 0.1, 0.5, 1, 5, 10, 20, 40, 60 μg/ml) of recombinant canstatin in the presence of 20 ng/ml of rhVEGF-A for 48 h. The cells were trypsinized and counted using a hemocytometer. Data are presented as mean ± S.D. of three independent experiments ($^{*}p < 0.05$, $^{**}p < 0.01$, $^{***}p < 0.001$).

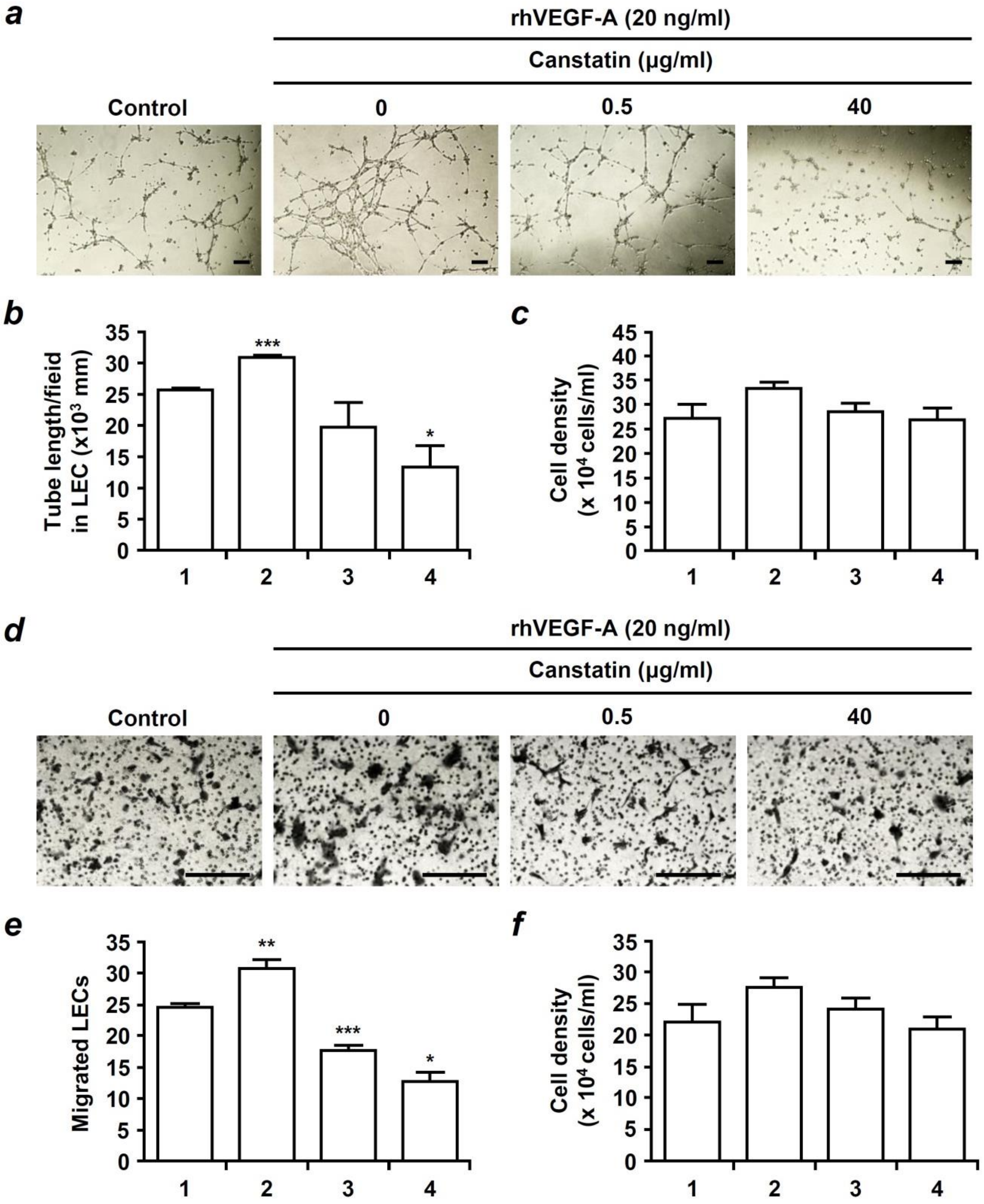

Supporting Information Fig. 2. Recombinant canstatin reduced the tube formation and migration of HLMECs stimulated with rhVEGF-A. (*a*) HLMECs in EBM-2 containing 2% FBS were added to Matrigel-precoated 48-well plates and treated with different concentrations (0, 0.5, 40 μg/ml) of recombinant canstatin in the presence of 20 ng/ml of rhVEGF-A. After 8 h of incubation, cells were imaged under a phase contrast inverted microscope using a digital camera. Scale bar = 200 μm. (*b*) Total tube lengths of HLMECs were quantified using the Image J program. (*c*) The cell densities of HLMECs under the same conditions as experiment (*a*) were determined after trypsinization, and are represented as a bar diagram. (*d*) HLMECs in EBM-2 containing different concentrations (0, 0.5, 40 μg/ml) of recombinant canstatin were added to the upper chamber of a transwell insert with 8.0 μm pore-sized polycarbonate membranes that were coated with 0.1% gelatin. EBM-2 containing rhVEGF-A (20 ng/ml) was added to the lower chamber to induce cell migration. After 24 h of incubation, cells that migrated to the undersides of the inserts were fixed with methanol, stained with Harris hematoxylin solution, and imaged under a phase contrast inverted microscope using a digital camera. Scale bar = 200 μm. (*e*) Five digital images per well were obtained in experiment (*d*), and the numbers of migrated HLMECs were counted. Each sample was assayed in duplicate. The numbers of migrated HLMECs present in 320 mm^2 are presented as a bar diagram. (*f*) The cell densities of HLMECs under the same conditions as in experiment (*d*) were determined after trypsinization, and are represented as a bar diagram. Data are presented as mean ± S.D. of three independent experiments ($^{*}p < 0.05$, $^{**}p < 0.01$, $^{***}p < 0.001$).

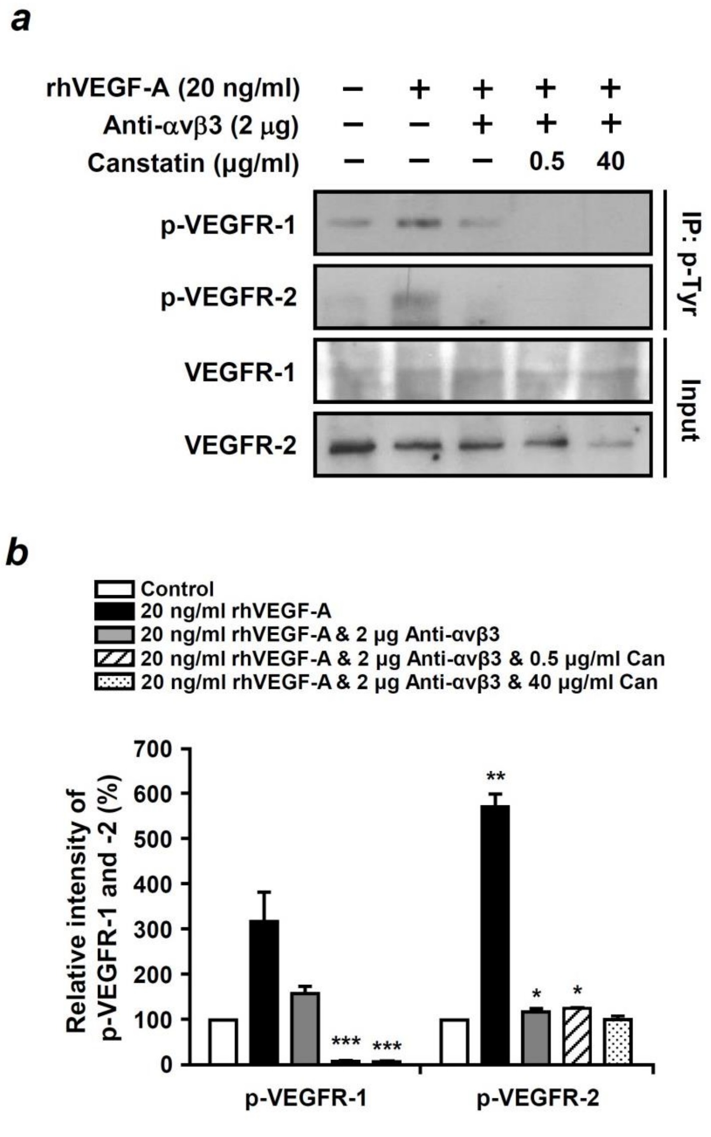

Supporting Information Fig. 3. The phosphorylation of VEGFR-1 and -2 by VEGF-A is reduced by the presence of anti-αvβ3 and recombinant canstatin. (*a*) HLMECs were treated with anti-αvβ3 (2 μg/ml) in the presence or absence of 20 ng/ml VEGF-A and recombinant canstatin (0.5 or 40 μg/ml). Cell lysates were immunoprecipitated with anti-phospho-Tyr (anti-p-Tyr) antibody. The presence of p-VEGFR-1 and p-VEGFR-2 in immunoprecipitates was detected via western blot analysis using anti-VEGFR-1 and anti-VEGFR-2 antibodies. (*b*) The amounts of p-VEGFR-1 and p-VEGFR-2 determined in three independent experiments of (*a*) were quantified and are represented as a bar diagram. The levels of the p-VEGFR-1 and p-VEGFR-2 of recombinant canstatin- and rhVEGF-A-untreated cells were established as 100%. Data are presented as mean ± S.D. of three independent experiments ($^{*}p < 0.05$, $^{**}p < 0.01$, $^{***}p < 0.001$).
